# Social change agent training program tailored to occupational therapists’ needs: a design-based study protocol

**DOI:** 10.1186/s12909-019-1530-1

**Published:** 2019-03-29

**Authors:** Sarah Rahimaly, Michaël Beaudoin, Denis Bédard, Anne Hudon, Emmanuelle Jasmin, France Verville, Annie Carrier

**Affiliations:** 1 School of Rehabilitation, Faculty of Medicine and Health Sciences, Université de Sherbrooke, 12e avenue Nord, Sherbrooke, Québec J1H 5N4 Canada; 2 Research Centre on Aging, University Integrated Health and Social Services Centre of the Eastern Townships – Sherbrooke University Hospital (CIUSSS de l’Estrie–CHUS), 1036 rue Belvedère Sud, Sherbrooke, Québec J1H 4C4 Canada; 3 Faculty of Education, Université de Sherbrooke, 2500 boulevard Université, Sherbrooke, Québec J1K 2R1 Canada; 4 School of Public Health and Health Systems, University of Waterloo, 200 University Avenue West, Waterloo, Ontario N2L 3G1 Canada; 5 Faculty of Law, Civil Law section, University of Ottawa, Fauteux Hall, 57 Louis-Pasteur Street, Ottawa, Ontario K1N 6N5 Canada; 6 University Institute of Primary Health and Social Services, CIUSSS de l’Estrie–CHUS, 1036 rue Belvedère Sud, Sherbrooke, Québec J1H 4C4 Canada; 7 Canadian Association of Occupational Therapists, 34 Colonnade Road, Suite 100, Nepean, Ontario K2E 7J6 Canada

**Keywords:** Social change agent, Occupational therapy, Training, Toolkit, Co-development, Iterative phases, Participatory process

## Abstract

**Background:**

As social change agents (SCAs), occupational therapists (OTs) are expected to defend the rights of their clients, advocate for and with them, and try to influence organizational and political decision-makers. However, OTs do not generally feel equipped to take effective action. The overall goal of this research partnership is to support practising OTs in acquiring the knowledge and skills required to act as SCAs through a specific SCA training program and a toolkit that summarizes the key training points.

**Methods:**

The study will include three iterative phases (conceptualization, implementation and evaluation) and use a participatory process. The design of the training program and toolkit will draw on the expertise of the researchers (theoretical knowledge), a professional provincial partner and study participants (experiential knowledge). To evaluate the training program and toolkit, a self-administered evaluation questionnaire, facilitator observation grid and semi-structured guide designed to facilitate focus group discussions will be used. The quantitative and qualitative data will be analyzed using descriptive statistics and thematic analysis, respectively. The results of the initial implementation and evaluation phases will inform improvement of the training program and toolkit before starting the cycle with the following groups.

**Discussion:**

In addition to training about 100 OTs, this study will produce three main benefits: 1) development of two products, namely the SCA training program and toolkit, that are easy to reuse and potentially transferable to other professionals; 2) ownership of these products by the partner through its close involvement in all stages of the study; and 3) development of a sustainable partnership between a team of researchers and a recognized organization with networks across Canada and internationally. These three spin-offs will provide a solid basis for an increasing number of permanent implementation initiatives, in Québec and elsewhere in Canada, not just in occupational therapy but also in other professions.

## Background

To support the social inclusion of people with disabilities, including participation in meaningful social roles and access to public services, occupational therapists (OTs) are key professionals [1]. As experts in human occupation [2], OTs work on a daily basis to promote the social inclusion of people of all ages with various disabilities, including neurological, physical and psychological disabilities [3]. Thus, OTs are prime witnesses of the systemic difficulties these people encounter. OTs’ interventions aim to abolish barriers [3], such as physical (e.g. adapting the urban environment), social (e.g. promoting access to employment), cultural (e.g. reducing ageism and other forms of discrimination) and institutional barriers (e.g. promoting access to public services). However, to do this, systemic and concerted actions (e.g. influencing decision-makers) are needed [4]. These actions are included in the role of social change agent (SCA) embodied by OTs.

In promoting occupational justice [1] and addressing the population’s social and health needs through the introduction of local and adapted changes [5], the role of SCA is part of OTs’ Practice Profile [6]. This Competency Profile guides the accreditation process for university occupational therapy programs in Canada [7] and provides a reference framework for occupational therapy best practices. According to this framework, as SCAs, OTs have a responsibility to promote the occupational needs and defend the rights of their clients (individuals and groups as well as populations), to advocate for and with them, and to try to influence organizational and political decision-makers [6]. Thus, to promote social inclusion, OT SCAs should analyze the organizational and sociopolitical context [8, 9], make use of communication strategies [9, 10] and systematically plan their actions [11, 12].

Communication has been highlighted as one of the many important and necessary skills of the SCA. According to the Competency Profile, OTs should be able to communicate adequately inside their system (as a competent agent) and outside their system (as a proficient agent) [6]. The literature suggests that SCAs should master good communication skills [12–14], both spoken and written [15], attentive listening [15, 16], partnership development and collaboration [14–17], and be able to plan, implement and evaluate their actions [5, 15, 16]. More specifically, efficient communication includes the ability to synthesize, integrate and transfer knowledge to decision-makers and organizational and political actors as a strategy to persuade them that the desired change is legitimate [10, 16]. Authors discussing SCAs’ required skills also emphasize reasoning abilities, which refer to the capacity to understand the context in which the change takes place [17, 18], including the actors and stakeholders, as well as the political strategy [12] and reasoning in an organizational state of mind [18].

The knowledge and skills SCAs require therefore differ from the clinical skills taught in entry-level graduate programs for OTs [12]. The knowledge and skills clinicians require focus mainly on clients and their neurological, physical and psychological abilities, along with dimensions of their environment, the analysis of occupational activities, occupational therapy assessments and interventions [19]. The SCA role, on the other hand, requires OTs to master political literacy, argumentation [12], strategic thinking and communication, uniting people around a project and assuming leadership [15]. In light of these discrepancies, more explicit integration of the SCA role in university and continuing education programs has been suggested to better help OTs fill this important role [20].

Despite the importance of the SCA role, OTs do not feel equipped to take effective action [15, 21]. In an electronic survey of 1196 OTs in Québec (Canada) who had recently completed a master’s degree in occupational therapy (average 3.6 years ±1.9), only 27.4% of the respondents felt competent to take on the role of SCA [22]. Considering that not all the competencies related to this role are taught in four of Québec’s five university programs [23–26] and that only 24.9% of OTs in Québec have a master’s degree in occupational therapy [27], this percentage is probably lower among practising OTs in Québec as a whole. In fact, a mixed-methods study of practising Canadian OTs showed that, although the respondents thought the SCA role was essential to meet their clients’ needs, they did not think they possessed the knowledge and skills necessary to take effective action [21].

This perceived inability to act as a SCA could be attributable to the nature of OTs’ entry-level training. To date, only one occupational therapy program in Québec appears to formally include multidisciplinary SCA knowledge and skills in its curriculum [24], and this content is given through elective courses in other programs. None of Québec’s OT programs appears to include training for the role of SCA specific to the profession. This may be because entry-level OT programs are already packed with content, as are the curricula of other professions [28]. Moreover, we were not able to identify any continuing education programs for OTs in French, Québec’s official language.

Bearing all this in mind, supporting OTs to act as SCAs is one of the priorities of the Canadian Association of Occupational Therapy (CAOT) [29] and its Québec Chapter (CAOT-Qc). CAOT is a professional association whose mission includes promoting the leadership of OTs in order to facilitate their clients’ participation in significant social roles. As part of this mission, CAOT-Qc has set itself the specific objective of providing tools and opportunities for Québec’s OTs to confidently act as SCAs. Accordingly, it surveyed Québec OTs in 2017 and found that OTs were motivated to act as SCAs with their respective clienteles but also that they needed help to do so. Specifically, OTs would like to know how to plan and conduct a SCA action process; this may include accurately describing the specific needs of a group of patients, analyzing the organizational and sociopolitical context, setting realistic and measurable goals, choosing effective strategies, planning and implementing them according to the target audience and measuring their effects. They would also like to master the analytical and communication skills required to complete the process. However, CAOT-Qc does not have the necessary knowledge or resources to produce and disseminate this knowledge and help develop these abilities independently. Since training programs for OTs should be both evidence-based and tailored to their specific needs, the contribution of researchers with expertise in pedagogy and the SCA role was perceived as necessary. Hence, CAOT-Qc partnered with the research team to create a continuing education program designed to achieve their priority goals for OTs. These priorities included OTs being able to understand the process for planning SCA actions, develop analytical and communication skills, and be ready to take concrete SCA actions.

### Objectives and conceptual frameworks

Based on recognized pedagogical principles and currently available evidence, the overall goal of this research partnership is to support OTs in acquiring the knowledge and skills required to act as a SCA. The specific objectives are to: 1) develop a SCA continuing education program and toolkit, 2) implement them, and 3) evaluate them according to the participants’ perceptions of acquiring the knowledge and skills required to act as a SCA.

**Fig. 1:**
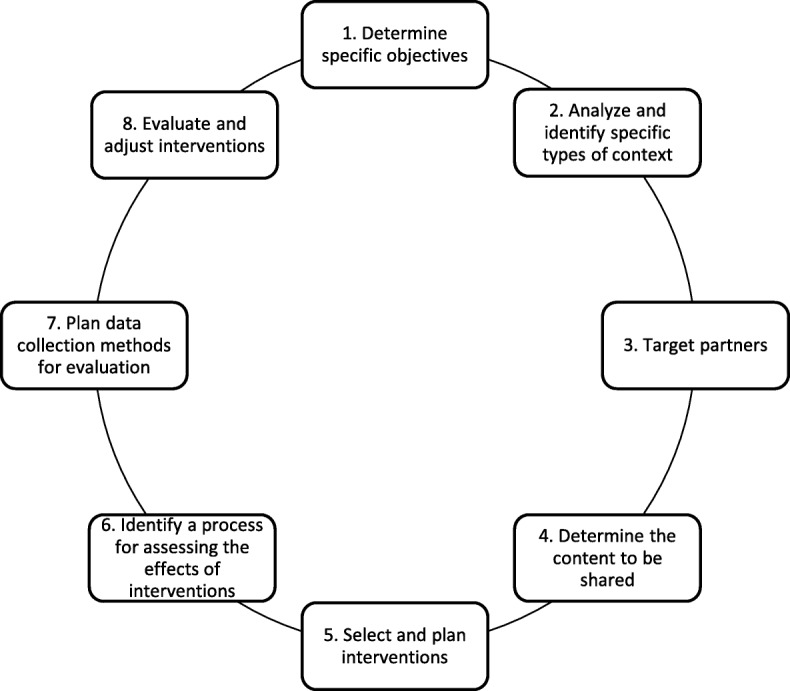
SCA Systematic Action Planning Model

Two theoretical frameworks support the conceptualization and operationalization of this approach. The first, the SCA Systematic Action Planning Model [11], describes a systematic process for analyzing and planning SCA actions (Fig. 1). This model was developed to help health professionals play the role of SCA and utilizes the skills required for this role and the results of a targeted literature review of: (1) intervention/ evaluation models, (2) intervention contexts, and (3) communication strategies. The model has eight iterative and dynamic steps. Its application is not prescriptive: it is intended to be flexible and responsive, two essential aspects of any SCA action. The second theoretical framework used is the Miller Competency Framework [30], which structures the acquisition of the knowledge and skills required for a specific role in four progressive levels of complexity, i.e. the person 1) knows, 2) knows how, 3) shows how, and 4) does. In this study, levels 1 and 2 will be specifically targeted. Both will be useful in the development of the training program and toolkit (Obj. 1) and their evaluation (Obj. 3).

## Methods/design

To achieve the study objectives, a cyclical, iterative and participatory process involving design-based research [31] will be deployed. Such a process makes it possible to develop a training program and adapt it rigorously and systematically by combining the expertise of researchers and field actors (here, the partner and OTs involved) in a real situation and by employing successive and iterative phases of conceptualization, implementation and evaluation [32] (see Table 3 in the Schedule section). These three phases are described in the Conduct of the Study section.

By mobilizing researchers in the fields of pedagogy and rehabilitation (theoretical knowledge), OTs (experiential knowledge), as well as an associative partner at the stage of identifying needs, this innovative project takes a cross-sectoral, co-constructive approach. Using the iterative and participatory process chosen, this strategy will ensure the relevance of the products developed by considering the needs expressed by CAOT-Qc and OTs (conceptualization phase) and feedback from OTs (evaluation phase).

### Data collection and participants

#### Conduct of the study

##### Conceptualization

Using Miller’s theoretical framework [30], this phase focuses on the training needs already identified by FV and CAOT-Qc OTs. After clarifying the definition and implications of the SCA role, the initial content of the training program and toolkit will include four specific themes: SCA action planning, contextual analysis, communication strategies and partnership development. The pedagogical method used will be the professional co-development approach [33], where three participants in turn occupy the role of client and the rest of the group acts as consultants [Table 1]). This approach provides the opportunity to leverage the participants’ experiential knowledge and foster reflection and peer modeling. The initial toolkit is a checklist that summarizes the important theoretical content for carrying out SCA actions (Table 2); it synthesizes the four training themes in a visual form on two double-sided 8½-X-11 sheets. It includes blank spaces so the would-be SCA will be guided to contextualize the different concepts from the training program and apply them to the situations in which they want to take action.

**Table 1:** Pedagogical method and process

Pedagogical method	The teaching method used, i.e. professional co-development, is a “training approach for people who believe they can learn from each other in order to improve and consolidate their practice. The reflection carried out, individually and in groups, is factilitated by a structured consultation exercise that addresses problems currently experienced by participants” [42].
Roles	- “Client”: has a change objective for which help is needed - “Consultant”: helps the client using own experiences - “Facilitator”: acts as a guide through the different steps and is responsible for the theoretical content of the session
Process	Training steps: 1.The professional co-development pedagogical approach will be explained to the participants. 2.Depending on the size of the group, participants will be divided into teams of about 6 to 8 people. 3.Co-development steps a. Presentation of the situation requiring the change: The problem is a situation to be changed where SCA actions are needed. The client explains the situation, the consultants listen. b. Clarification: The consultants ask questions; the client responds and clarifies. c. Contract: The client formulates the request to the group and specifies the type of consultation desired. The consultants and the client together ensure that the contract will allow consultation. d. Consultation/exploration: The consultants react. They share their impressions, reflective questions, reactions, comments, ideas, suggestions, etc. The client listens without debate, clarifies if necessary, and makes note of the consultants’ suggestions. e. Summary of learning and action plan by the client: The client assimilates the information, indicates what is retained and designs an action plan. At this stage, the consultants summarize what they have learned that day. f. Learning and regulation: The client and the consultants describe what they have learned. They regulate themselves and evaluate the session. 4.During the discussions, the facilitators (SR, MB and FV) will ensure that the themes mentioned (theoretical content) are addressed at the appropriate times in the various stages of the co-development process. 5.This sequence will be repeated three times to explore three different situations.

**Table 2:** Toolkit content

Content	- SCA role definition reminder - Contextual analysis (internal and external contexts) - Communication strategies - Partnership development (partnerships and stakeholders)

##### Implementation

Following ethics approval, FV will email OT members of CAOT-Qc interested in participating in the training program and evaluation. These OTs belong to six pre-existing groups (four in Montréal, one in Québec City and one in Sherbrooke; *n* = between 12 and 50 OTs per group), based on their different clients (e.g. children, adults) and intervention communities (e.g. school, local community service centre). The recruitment strategy will be broadened by posting the invitation on the CAOT-Qc website and including a link to the page via CAOT-Qc Facebook and Twitter accounts. Interested OTs will be invited to contact FV if they have any questions. FV will relay the contact information of the interested participants to MB and SR, who will be responsible for calling each participant and ensuring that the objectives and overall study are explained and understood, and that all questions pertaining to the study are answered. Potential participants who are still interested will be sent the consent form by email or mail, at their discretion. All interested OTs and CAOT-Qc members will be eligible to participate in the training. Once free and informed written consent has been obtained (see Ethical Considerations section), recruited participants will be asked to respond to a Doodle to identify a common training date for their group outside of working hours. FV, MB and SR will deliver the training session and present the toolkit to the OT participants (Obj. 2), one group at a time.

##### Evaluation

To evaluate the training program and toolkit (Obj. 3) and adapt their design accordingly, the team developed various tools, which were validated by two methodological and content experts. These tools are a self-administered evaluation questionnaire, facilitator observation grid and semi-structured guide to facilitate focus group discussions. First, immediately before and after the training session, all participants will complete a self-administered evaluation questionnaire. The facilitators (FV, MB and SR) will complete an observation grid. A convenience sample of 5 to 8 OTs will be invited to participate in an audiotaped focus group meeting lasting 60 to 90 min. To identify and recruit these participants, a separate sheet will be included with the self-administered questionnaire specifically asking participants about their interest in participating in a focus group meeting sometime after the training session. The meetings will be held outside working hours and the discussion will be facilitated by a trained research assistant with 10 years of experience in group animation and pedagogy. To ensure that, on the one hand, participants have enough time to reflect on the training program and toolkit – specifically, how they met their needs as SCAs – and that, on the other, those reflections are still fresh in their minds, the focus group meeting will take place approximately 1 week after the training session. After each training session, the collected data will be analyzed (see Analysis section) and the results will be considered with a view to adapting, modifying or improving the next training session and toolkit before starting the Implementation-Evaluation cycle with the next group. After this analysis, the evaluation tools will be reviewed. To document the process, a logbook will be completed by MB and SR, with the support of the team.

### Research tools

#### Self-administered evaluation questionnaire

This form includes three sections: pre-training, post-training and sociodemographic data. The pre-training section includes questions about the participant’s intended goals and perceived ability to act as a SCA. Derived from the Participant Workshop Evaluation assessed by Dagenais et al. [34], the post-training section includes questions about each activity relating to the acquisition of knowledge and skills required to act as a SCA. For example, the participants will be able to indicate their degree of satisfaction with different aspects of the training, such as the content of the training program and toolkit, the activities, the available material, the duration, the facilitators, and the overall experience. They will also be asked to indicate if they feel competent to act as a now-trained SCA. Finally, the sociodemographic section characterizes the participants’ profiles (e.g. age, clinical setting, clientele served, years of experience). The questionnaire will be available in two formats: electronic and paper. When possible, wifi access will be provided during the session for the participants to complete the questionnaire. The electronic questionnaire will be hosted on LimeSurvey software because of the easy extraction of data into Excel files.

#### Facilitator observation grid

The facilitator observation grid highlights the elements to be improved and/or maintained with respect to the training process and participation in the different activities (e.g. ease of carrying out activities, achievement of objectives, time required, etc.). Due to the iterative nature of this study, the observation grid will be an important tool for helping to improve the training over time.

#### Semi-structured guide to facilitate focus group meetings

Using open-ended questions, the focus group will discuss the themes from the evaluation form in order to generate in-depth feedback. For example, “Training participants’ perceived increase or decrease with respect to knowledge, skills or confidence between the beginning and the end of the training. What do you think this increase/decrease is attributable to?” Elements of the facilitators’ observations (e.g. degree of commitment to the training activities perceived by the participants) could also be validated or expanded upon with the group.

#### Logbook

The logbook completed by the research trainees will track the progress of the study, changes to the training program and toolkit, and areas for improvement according to the team. It will also be used to note the methodological choices made and any preconceptions influencing the team during the analyses.

### Data analysis and interpretation

The data from the evaluation form, observation grid and focus group meeting will be compiled and transcribed. The quantitative data from the form will be analyzed using descriptive statistics. The qualitative data will be analyzed thematically by at least two members of the team. First, saliency analysis [35] will be used to identify emerging themes according to each unit of meaning. Then, for each theme identified, this analysis technique will lead to the attribution of conceptual importance and recurrence. The importance of the themes will be determined by whether it is possible to operationalize them to improve the training program and toolkit, and recurrence will be determined by their frequency of occurrence. Finally, all of the participants’ data will be triangulated with the facilitators’ observations and the results interpreted. To increase the rigor of the process and facilitate the exploration of alternative interpretations, the analyses and interpretation of the results will be discussed and validated by all members of the team. Finally, from the analyses carried out and the logbook, the innovative pedagogical process will be modeled to facilitate its transferability to similar contexts. To do this, the team will draw on logic model elements [36]: components and activities, target groups, results, inputs, outputs, contributing and external factors.

### Role and contribution of the study partner

In addition to participating in developing the research protocol, CAOT-Qc will provide logistical and technological support (e.g. teleconference) to facilitate interactions between research team members and group leaders. CAOT-Qc will also be very active in the recruitment, design, organization and facilitation of training for different groups. To this end and as required, CAOT-Qc will offer the use of its material and information resources for teleconferences, webinars, and hosting interactive training modules, including video capsules, PDF or other documents. In addition, CAOT-Qc intends to play a significant leadership role in the dissemination of the project and its results.

### Strengths and limitations

First, this research partnership will provide OTs with a long-term opportunity for professional development based on available evidence concerning SCAs. More specifically, this project will provide professionals with highly contextualized training on the role of SCA, a socially important role that is currently under-invested in. It will also lay the groundwork for creating more opportunities to implement the training program by a multiplying agent (FV). Combining knowledge from various disciplines (occupational therapy, pedagogy) and using participatory research methods, this project will be a unique training opportunity for two research trainees. Finally, this innovative process will contribute to the development of knowledge regarding cross-sectoral participatory research and may be used by other groups to increase the flexibility of their own training process relating to complex roles. Methodological choices will ensure the scientific rigor of the study and make it easier to obtain rich data through in-depth focus group meetings and prolonged field exposure (data collection over more than a year) [37]. The professional experience of the team is a strength since it makes them more sensitive to the perspective of OTs [38]. Moreover, given their proximity to the research object, members of the team will pay particular attention to their assumptions and preconceptions throughout the study. The use of a logbook and triangulation of the researchers’ perspectives will also ensure rigor in the analysis and interpretation of the results [38]. Social desirability [39] is a potential bias in the post-training evaluation process, including the questionnaire and focus group discussions. The effects of the researcher on the site and the site on the researcher [40] should also be considered since the team knows the OTs potentially involved in the study. This could influence data collection negatively (self-censorship of participants, biased observations of researchers) or positively (greater trust, richer data).

### Ethical considerations

The research protocol has been accepted by the Research Ethics Committee of the CIUSSS de l’Estrie–CHUS. A consent form outlining the purpose of the study and potential benefits and risks will be presented to each of the participants. All participants will need to give their free and informed written consent [41]. Anyone can withdraw from the study at any time, without consequence. Participants will also be informed of the confidential nature of the data collected and the procedures followed to ensure this confidentiality and their anonymity. In particular, the data collected, electronically or on paper, will be locked (principal investigator’s work computer or flame-retardant binder at the Center for Research on Aging), and only the research trainees (MB and SR) and principal investigator (AC) will have access to it. To limit any risk to the confidentiality of answers to the self-administered questionnaire, information concerning interest in participating in the focus group and the contact details of interested persons will be collected on a separate sheet.

## Discussion

This project will develop the role of OTs as SCAs which, despite its importance and presence in the Competency Profile [6], remains underutilized because OTs lack the necessary knowledge and skills. In addition to training about 100 OTs to act as SCAs, this study will produce three main benefits: 1) development of two products, namely the SCA training program and toolkit, that are easy to reuse and potentially transferable to other professionals; 2) ownership of these products by the partner through its close involvement in all stages of the project; and 3) development of a sustainable partnership between a team of researchers and a recognized organization with networks across Canada (CAOT) and internationally (World Federation of Occupational Therapy). These three spin-offs will provide a solid basis for generating an increasing number of permanent implementation initiatives, in Québec and elsewhere in Canada, in both occupational therapy and other professions. In this respect, the modeled experience will provide useful insights for other groups undertaking similar cross-sectoral participatory training approaches.

### Dissemination of results

In addition to the traditional methods (two articles, to be submitted to medical education journals and presented at conferences), forums with knowledge users (OTs and their clients) will be organized in more remote regions of Québec by FV with the support of AC, EJ and CAOT-Qc’s logistical infrastructure. These forums will stimulate regional OTs’ interest in the training program and, hopefully, its broader implementation. To do so, these forums will discuss the role of an “effective SCA” and the usefulness of the training program and toolkit in addressing the barriers to social inclusion reported by OTs and their clients in light of the research findings.

In addition, CAOT-Qc will present the results of the study to the board of directors of the national organization (CAOT). The findings will also be disseminated to CAOT’s 16,000 members, OTs and students via *OTWeekly* (CAOT’s email newsletter), as well as to the Alliance of Canadian Occupational Therapy Professional Associations (ACOTPA), Association of Canadian Occupational Therapy University Programs (ACOTUP) and various partners (e.g. patient advocacy groups, associations of other professionals) through work meetings. CAOT-Qc gives the team access to its information technology resources (website, Facebook profile, Twitter, *OTWeekly*) for advertising purposes: the information will be widely disseminated and easily accessible.

### Schedule

The study began in May 2018 with the review and synthesis of relevant literature and development of the protocol, including collection tools. Ethics approval was obtained from the Research Ethics Committee of the CIUSSS de l’Estrie–CHUS on 23 July 2018. The data collection and analysis will take place from January 2019 to March 2020. Results will be disseminated from March to May 2020. Table 3 presents this schedule along with input from members of the team.

**Table 3:** Tasks, implementation period and contribution of team members in each phase

Tasks	Period	Contribution of team members							
		AC	SR	MB	DB	AH	EJ	FV	A
PLANNING									
Steps for ethics approval by the Research Ethics Committee	07/18	●		●	○	○	○		
Review and synthesis of relevant literature (pedagogy, role of SCA)	05/18–01/19	●		●	●	○			♦
Based on scientific data and good practices in pedagogy, determination of:									
• content of training program and toolkit		●	●	●	●	○	○	○	
• pedagogical methods used and form of the toolkit		●	●	●	●	○	○	○	
Preparation of:									
• training materials and toolkit (initial versions)		●	●	●	●	○	○	●	♦
• collection tools (observation grid, evaluation form and semi-structured guide, logbook, obj. 3)		●	●	●	●	○	○	○	♦
Recruitment of participants and logistical organization of training sessions		●	●	●				●	♦
IMPLANTATION									
Training session with each group	01/19–03/20	●	○	●	●			●	♦
EVALUATION									
Data collection (including logbook)	01/19–03/20	●	●	●	●			●	♦
Data processing, analysis and interpretation		●		●	●	○	○	○	♦
Modifications to the training program and toolkit according to the comments		●		●	●	○	○	○	♦
Adaptation of collection tools (following the first focus group meeting)		●		●	●	○	○	○	♦
Dissemination of results (articles, conferences, forums, information resources, new groups, team contacts)	03–05/20	●	●	○	●	○	●	●	♦

A: Research assistant●: Team member responsible for carrying out the task and/or supervising research trainees; ○: Contribution (feedback, validation); ♦: Technical support

## Abbreviations

ACOTPA: Alliance of Canadian Occupational Therapy Professional Associations
ACOTUP: Association of Canadian Occupational Therapy University Programs
CAOT-Qc: Canadian Association of Occupational Therapists Québec Chapter (English equivalent of *Association canadienne des ergothérapeutes – Québec*)
CIUSSS de l’Estrie–CHUS: *Centre intégré universitaire de santé et de services sociaux de l’Estrie - Centre hospitalier universitaire de Sherbrooke*
OT: Occupational therapist
SCA: Social change agent
